# Use of high-content analysis and machine learning to characterize complex microbial samples via morphological analysis

**DOI:** 10.1371/journal.pone.0222528

**Published:** 2019-09-23

**Authors:** Jennifer Petitte, Michael Doherty, Jacob Ladd, Cassandra L. Marin, Samuel Siles, Vanessa Michelou, Amanda Damon, Erin Quattrini Eckert, Xiang Huang, John W. Rice

**Affiliations:** Novozymes North America, Inc., Durham, North Carolina, United States of America; University of California Berkeley, UNITED STATES

## Abstract

High Content Analysis (HCA) has become a cornerstone of cellular analysis within the drug discovery industry. To expand the capabilities of HCA, we have applied the same analysis methods, validated in numerous mammalian cell models, to microbiology methodology. Image acquisition and analysis of various microbial samples, ranging from pure cultures to culture mixtures containing up to three different bacterial species, were quantified and identified using various machine learning processes. These HCA techniques allow for faster cell enumeration than standard agar-plating methods, identification of “viable but not plate culturable” microbe phenotype, classification of antibiotic treatment effects, and identification of individual microbial strains in mixed cultures. These methods greatly expand the utility of HCA methods and automate tedious and low-throughput standard microbiological methods.

## Introduction

High Content Analysis (HCA) methods are widely deployed in the drug discovery realm and are utilized for phenotypic drug discovery, targeted drug discovery, and target identification and characterization[1]. Enabled by the commercialization of various HCA instruments beginning in the late 1990’s, these automated microscopic platforms have greatly expanded the ability to perform cell-based screening by reducing labor time and variability of manual microscopic analysis. The ability of HCA instruments to multiplex data types and associate numerous features to individual cells can easily generate large datasets, which allows for feature analysis on a massive scale utilizing advanced machine learning techniques[2]. Machine learning supports mutable algorithmic analysis of large data sets, without pre-defined data definitions. The raw data is iteratively analyzed, leading to increased accuracy of the resulting predictions[3]. Linking high-content imaging, single-cell analysis, and machine learning, researchers within drug discovery accelerate the process and quickly extract results from big data. However, within microbiology, HCA of individual cells has been slow to develop. We demonstrate here the power of HCA to progress beyond the traditional methods of colony forming units per milliliter (CFU/mL), selective media, and culturing pure strains, pioneered in the early 1900’s[4].

With the aid of various fluorescent dyes and biomolecules, direct microscopic observation methods and flow cytometry have transformed microbiology laboratory procedures[5–8]. These methods include the use of fluorescence *in situ* hybridization methods [9], along with Atomic Force and Raman microscopy[6,10]. There are also recently described methods allowing identification of *Giardia lamblia* using a cell phone application[11]. In parallel, large scale microbial research is becoming more prevalent. For example, first launched in 2007, with a second phase launched in 2014, the Human Microbiome Project has expressed goals of generating the resources and tools to fully characterize the human-associated microbiome (https://hmpdacc.org)[12]. An effort to characterize as much of the currently un-cultured microbial diversity as possible has also been launched, known as the Earth Microbiome Project[13]. Additionally, in agriculture, efforts are currently under way to study and gain insight into plant-microbe interactions[14–16]. With these vitally important and critical initiatives in progress, our ability to miniaturize and convert traditional microbial plating methods to high-throughput methodology has never been more important.

The efforts of our laboratory have been focused on this challenge, with a mandate of converting various bioassays and enumeration methods, traditionally using a 100mm petri dish, to formats capable of being fully integrated into high-throughput formats. Development of this process involved the incorporation of 96- and 384-well plate handling and assay formats, along with automated liquid handling robotic protocols. The work described here details our approach to incorporating traditional HCA methods into the methodologies, techniques, and data analysis workflow of a traditional microbial discovery laboratory environment. Specifically, we describe methods that facilitate high-throughput cell enumeration and determination of viability from microbial samples, including complex mixtures of strains, as well as the application of various machine learning analysis methods for the identification and classification of microbes in mixtures. Taken together, these techniques allow for faster data turn-around, greatly reduced physical resources, and high-throughput image-based characterization of microbial samples.

## Materials and methods

All cell stains and microbial media formulations were from Thermo Fisher. Cellcarrier 384-well, black wall, clear-bottom plates were from Perkin Elmer. All antibiotics were from Sigma-Aldrich. CLICK-IT^™^ Homopropargylglycine (HPG) 594 viability kits were from Thermo Fisher (cat #C10429), and Syntrix-25 material was from Noviocell. High-content images were acquired with the GE INCell 2200 high content platform. Microbe strain identification was performed using a Bruker MALDI Biotyper, following manufacture’s recommendations. Statistical and machine learning analyses were performed with JMP or JMP Pro, version 14.0, unless otherwise indicated.

### *Bradyrhizobium japonicum* plating and enumeration

To demonstrate cell adherence, *Bradyrhizobium japonicum* (USDA ACCES strain 110) samples were prepared by inoculating a shake flask of YEM broth with a single cell colony and incubating the flasks for 3–5 days at 30°C. At harvest, cell samples were prepared as described for enumeration. For enumeration of *B*. *japonicum* experiments, frozen fermentation samples were centrifuged at ~13,000 x g for 8 minutes to pellet the cells. Spent media was removed, and the cell pellet was resuspended in phosphate buffered saline (PBS). These samples were then either plated onto YEM agar plates for CFU/mL determinations or stained with a solution of 10μM SytoBC, 10μg/mL DAPI and 5μM Propidium iodide (PI) or 5μM SYTOX Orange, for approximately 1 hour. Stained cells were then serially diluted 1 to 5, with the starting concentration at 1:10 from the original (final dilutions 1:50, 1:250, 1:1250, and 1:6250) in PBS, and a 50μL sample for each dilution was transferred to a Syntrix-25 coated CellCarrier 384-well plate for imaging. Imaging plates were spun at 1,400 x g for 2 minutes, to adhere the bacteria cells to the plate surface. Following centrifugation, images for DAPI, FITC, and Cy3 filtersets were captured using an INCell 2200 high content imaging platform. Detection algorithms for individual object detection from the raw images were developed within GE Developer software (version 1.9.3), and an example detection algorithm is presented in the **Supplemental Material** (S5 Table) section. Cellular autofluorescence intensity values in the Cy3 spectrum, used as a background signal cut-off value for PI and SYTOX Orange intensity levels, were determined using cell samples that were heat killed at 70°C for 30 minutes prior to staining. These cut-off values were calculated differentially for each strain. Using the combined signal from the DAPI and FITC channels to describe the individual *Bradyrhizobium* cells, viability was determined using PI or SYTOX Orange staining intensity, above cut-off, to identify non-viable cells. Formulas used to determine the cell counts persample are described in the **HCA Enumeration Calculations** section in this manuscript. For the CFU/mL assays, agar plates of cells were incubated for 5 days at 30°C, and discrete colonies were counted for each dilution. All reported cell counts are average values from three replicates, for each sampling dilution that demonstrated linearity, described as cell counts per dilution with an R^2^ value of >0.95.

### Comparison of enumeration methods

For each strain tested, 300μL from a frozen stock was used to inoculate a 50mL conical tube with filter cap containing 15mL of a nutrient rich media. This pre-culture was grown overnight with shaking at 225 RPM, and 30°C. The pre-culture was transferred to a 2.5L shake flask, containing 700mL of a nutrient rich media, and grown overnight with shaking. Flask cultures were harvested by centrifugation (15,000 x g, 10 min, 4°C) where the supernatant was removed until a 5x concentration was achieved. Fifteen percent glycerol (w/w) was added to the samples, followed by mixing and removal of 100μL for enumeration. These samples taken from each fermentation were sequentially diluted 1:10 in PBS, in triplicate. One hundred microliters of the serial diluted samples, corresponding to 1x10^-6^, 1x10^-7^, and 1x10^-8^, were plated onto tryptic soy agar plates, resulting in final CFU/mL dilution plates of 1x10^-7^, 1x10^-8^, and 1x10^-9^. The same dilution samples were stained for flow cytometry enumeration with a Stratedigm S1000EXi Flow Cytometer. Samples were stained with 2μM of SytoBC and 1.25μg/mL of Propidium Iodide. After staining, the samples were run at a flow rate of 0.8μL/s. Enumeration was based on the acquisition time needed to collect 10,000 individual objects. The same original dilution samples used for CFU/mL plating were also used for HCA enumeration, using the method described for *B*. *japonicum* enumeration. Each analytical method had its result values averaged, and all presented statistics were performed on those average values using JMP (version 14). Three microbe samples did not generate any data from the flow cytometry enumeration method due to the samples clogging the flow cells, so these strains were not included in statistical analysis comparing the enumeration methods.

### Measurement of microbe total protein synthesis via CLICK chemistry

Individual microbe samples taken from biological isolations were collected in M9 minimal media. Using a Beckman Coulter FXp robot, microbe samples were transferred to a polypropylene V-bottom 96-well plate at 135μL/well, with four individual wells per microbe sample. Fifteen microliters of M9 media, containing 500μM HPG, was added to two of the four wells, giving afinal HPG concentration of 50μM. Fifteen microliters per well of M9 was added to the remaining two wells, to create corresponding replicates with and without HPG (+/- HPG). The V-bottom microbe test plates were then incubated for7 hours at 30°C, to allow for HPG to be incorporated into the global cellular protein pool. HPG incorporation enables determination of microbe viability via detection of total cellular protein synthesis, as non-viable microbes do not synthesize protein. At the end of this incubation, the V-bottom sample plates were centrifuged at ~3,000 x g for 7 minutes. Media was carefully removed, and the cell pellets were re-suspended in 95% methanol/5% acetic acid (v/v) for fixation.

Following fixation, +/- HPG samples were washed with PBS containing 0.01% Tween 20 and 0.25% TX-100, then processed following the CLICK-IT protocol provided by the manufacturer to attach a Alexa Fluor 594 azide to all HPG molecules incorporated into the total cellular protein pool. For identification of individual microbe cells, cellular DNA was stained with 5μM SYTOX Green. Once all sample processing was completed for the HPG incorporation, samples were transferred to Syntrix-25 coated 384-well CellCarrier plates, followed by centrifugation at 1,460 x g for two mintues. Images were then collected using the 100x objective on a GE INCell 2200 high content analyzer. These images were analyzed with GE Developer software, using a custom algorithm, based on individual object detection using the FITC channel (SYTOX Green stained DNA) to define each cell. An example detection algorithm is presented in **Supplementary Materials** (S6 Table). To determine HPG incorporation, individual objects from the duplicate wells from both +HPG and -HPG incubations were compared for levels of Alexa Fluor 594 staining intensity collected in the Cy3 imaging channel. Cellular auto-fluorescence intensity was determined differentially for each strain using the individual object intensity distribution in -HPG samples. The cut-off value was set at the 90^th^ percentile, to account for debris and outliers. Along with total cell counts, viable cell intensity values were determined. As described in **HCA Enumeration Calculations**, viable cells were identifiedby measuring the fluorescent signal in +HPG incubated cells above the cut-off, Correction for the 90^th^ percentile distribution is also shown in calculations. All calculated data was generated using JMP (version 14).

### Object identification of individual microbial morphologies following antibiotic treatments

Tetracycline (10mg/mL) and vancomycin (6mg/mL) stocks were prepared in sterile water. Individual colonies from each test microbial strain were selected from agar plates and used to inoculate a 35mL shake flask of Reasoner’s 2A (R2A) broth media, which were grown overnight at 30°C with 200 RPM shaking. Two of the three strains used, *Escherichia coli* (catalog number 10798) and *Pseudomonas fluorescens* (catalog number 53958) were obtained from ATCC, the *Bacillus megaterium* was isolated from soil samples and its identity was confirmed via Bruker biotyping. Following the overnight incubation in a shake flask, the *E*. *coli* and *B*. *megaterium* were diluted 1:20 into fresh medium, and the *P*. *fluorescens* was diluted 1:100. Diluted microbes were then transferred into sterile 96-well V-bottom polypropylene plates, to a volume of 100μL/well. Antibiotic stocks were diluted to 2X treatment concentration in R2A media, and 100μL/well of this was added to the 100μL microbe samples in the V-bottom 96-well plates. These treatment plates were then sealed with a Breath-Easy plate seals (Z380059, Sigma-Aldrich) for evaporation control, and incubated for an additional 18 hours at 30°C without shaking. Following this overnight incubation, 100μL/well of 10% buffered formalin was added to all plates for 30 minutes, followed by centrifugation at ~3,000 x g for 7 minutes. After centrifugation, supernatants were removed, and the resulting cell pellets were mixed with 150μL/well of PBS containing 0.01% Tween 20, 0.25% TX-100, and 5μM of the DNA stain SYTOX Green. Following sample preparation, the antibiotic treated cells were diluted in PBS, and transferred to Syntrix-25 coated 384-well plates, centrifuged for 2 minutes at 1,400 x g, and imaged with the GE INCell 2200 high content analyzer 100x objective.

The resulting images were analyzed using GE Developer software to determine various morphological and staining intensity values, with individual objects (cells) identified via DNA staining. Algorithm settings are presented in the Supplemental Data section. Since microbes, as prokaryote organisms, do not contain a nucleus, we generally observed DNA staining throughout the cell, as opposed to localized DNA staining usually associated with discrete nuclei in mammalian cells. Measures generated from individual objects were analyzed in JMP (version 14) and used to determine the effects of each antibiotic treatment on the microbial cultures. Unsatisfactory objects were excluded from analysis if they showed an end node value greater than 3. As defined within the GE Developer analysis software, end node is a fiber related measure, and we noted that objects with measured end nodes greater than 3 tended to be un-differentiated clumps or debris.

### HCA enumeration methods, calculations and machine learning applications

All HCA images were collected using a GE INCell 2200 high content fluorescent imaging platform, with a 100x/0.9 Plan Fluor objective. For image acquisition, typical exposure times were 0.3–0.8 seconds, using 1x1 binning and 2-D image settings. For imaging, all samples were serially diluted in PBS or PBS + 0.01% Tween 20 from original treatments. Given the generally unknown starting concentrations for each sample, this facilitated generating images that would contain as many discrete objects as possible, as opposed to images with objects too dense and overlaying each other. All images were analyzed using GE Developer software, image analysis protocols are presented in the **Supplemental Materials** section (S5 and S6 Tables), and all data outputs were based on individual object data.

**For the *B*. *japonicum* and enumeration method comparison experiments, total cell counts and total viable cell counts obtained from the INCell 2200 were determined formulas as follows**:

Totalcellspersample=totalobjectcounts*72.2*dilutionfactor*samplingfactor

Totalviablecellspersample=totalgatedobjectcountsaboveautofluorescentbackground*72.2*dilutionfactor*samplingfactor

The 72.2 value represents the total area imaged from each well in the 384-well imaging plates (9 fields) from a total possible number of images/well (equal to 650). Dilution factor refers to which final sample dilution was imaged. To correct for sampling volume, since each imaging sample was from a 50μL total volume sample, a factor of 20 was included to calculate the total cell counts/mL. For enumeration, we attempted to collect data from at least 3 separate dilutions, and only used data from wells with counts that represented a linear dilution with an R^2^ value of >0.95 for cell number totals.

**For viability determination based on HPG incorporation into total cellular protein synthesis, calculations used were**:

$$\begin{array}{l} Where\;A\;is\;the\;set\;of\;objects\;in\;the\;+HPG\;sample,\;and\;n\left(A\right)\;is\;the\;number\;of\;objects\;in\;set\;A,\;and\;a\;is\;a\;member\;of\;set\;A\\ And\;B\;is\;the\;set\;of\;objects\;in\;the\;corresponding\;-HPG\;sample\;A\\ And\;modedensity\;is\;the\;HCA\;measured\;mode\;density\;value\;on\;a\;by-object\;basis\\ P_{90}\left(B_{modedensity}\right)\;=\;x \end{array}$$

**Then**:

$$\Sigma \;[n(A)where\;a_{modedensity}>x]={n(A)}_{v},\;which\;is\;the\;number\;of\;viable\;objects\;in\;set\;A$$

TotalCells∀sample=n(A)*dilutionfactor*samplingfactor*72.22

$$Viable\;Cells\;\forall \;sample\;=\;(n(A)–({n(A)}_{v}–\;0.1*n(A))\;*\;dilution\;factor\;*\;sampling\;factor\;*\;72.22$$

For microbial identification from GE INCell image-based by-object feature data, we used machine learning methods provided by JMP Pro (version 14). Specifically, the bootstrap random forest machine learning algorithm with default settings. This platform predicts a response value by averaging the responses over many decision trees. Untreated control sample by-object feature data from the antibiotic treatment experiment were used to train the algorithm to detect each microbe type, with 60%/20%/20% training/test/validation partitions. Following training, by-object feature data from each training set was re-analyzed to establish an accuracy rate, then untreated control samples of mixed microbe strains were analyzed, and the random forest model created a most probable strain identification for each individual object.

## Results and discussion

**Fig 1:**
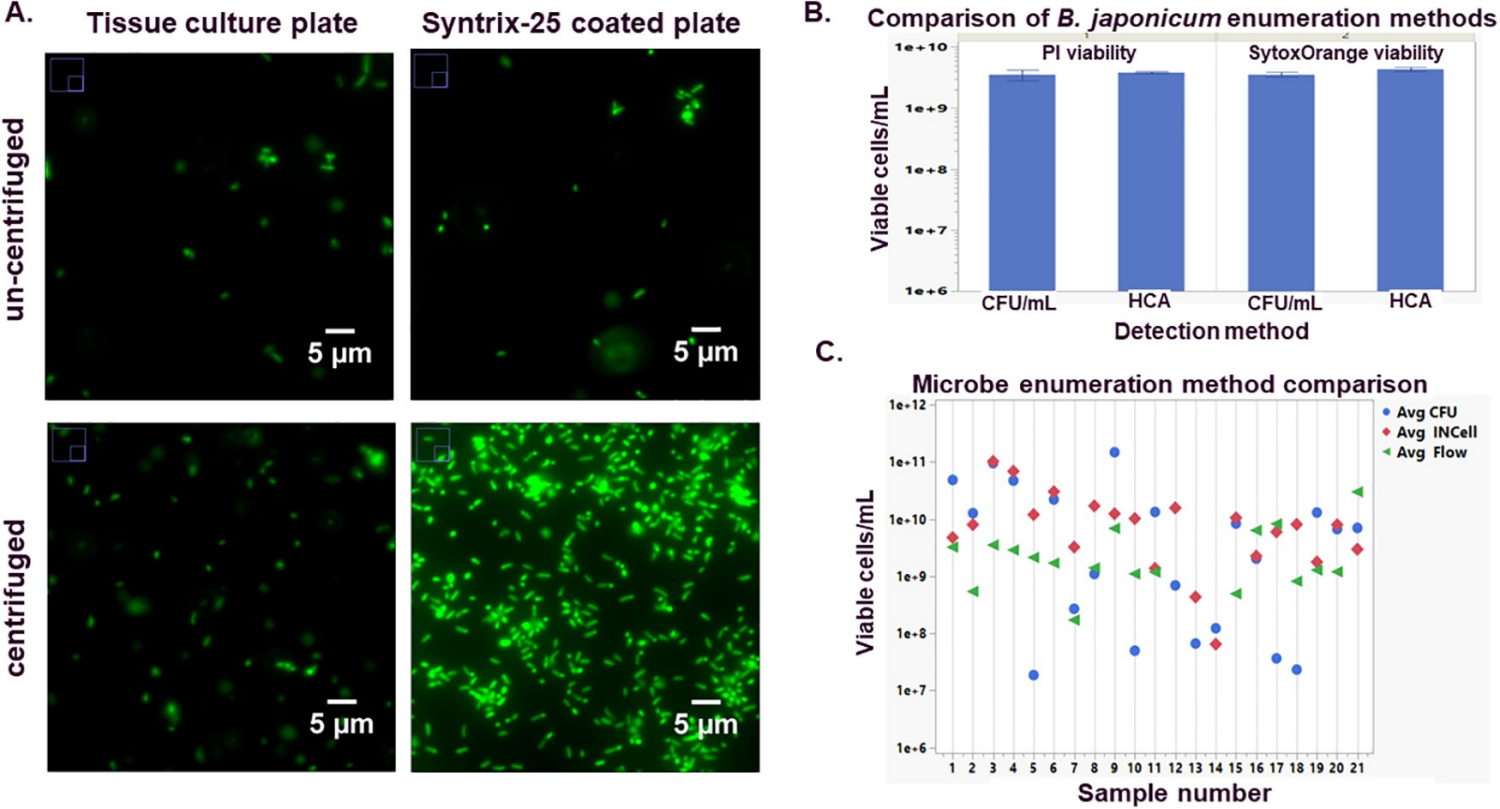
Microbial enumeration using HCA methods. Example images from SytoBC stained *Bradyrhizobium japonicum* are presented in panel **A**. For comparison, images obtained from un-coated, standard tissue culture plates are presented with images obtained from plates coated with Syntrix-25. To demonstrate HCA-enumeration methodology using a specific microbe, we utilized the *B*. *japonicum* strain, graphed data in panel **B**., represent the average of 3 separate samples taken from two separate cryopreserved samples from the same fermentation, error bars indicate the standard deviation. Test 1 viability was measured with PI, for test 2 we used SYTOX Orange to determine viable cells. Panel **C**. is the graph of average values obtained from 21 different microbial fermentation enumerated via CFU/mL, HCA, or Flow. All values are the mean of triplicate measurements.

Our initial efforts were focused on the practicality of using an HCA instrument to image microbes. Although direct microscopic observation is common in microbiology, utilizing a multi-well plate as the sample holder vs. a glass slide and cover slip presented the simple challenge of getting the cells in a flat, planar location to obtain in-focus images. Although we have utilized z-stacking and max-projection techniques to help gather object information, we wanted to maintain the speed of single image collection inherent with the collection of a simple 2-D image. This demanded that we use a method to capture and hold our cells stationary and flat during plate imaging. We approached this technical issue by exploring various plate coatings commonly used for cellular adherence. Much research has focused on the utilization of proteins or organic molecules to facilitate biomolecular interactions to allow for cellular adherence in multi-well plates[17–19]. We focused on commercially available coatings. After testing collagen, poly-lysine, and a proprietary coating known at the time as artiCYT (now marketed under the trade name Syntrix-25) we chose Syntrix-25 coated 384-well plates for protocol development. The Syntrix-25 coated plates demonstrated good cellular adhesion of the test microbes (Fig 1A) as compared to a non-coated, tissue culture-treated plate or plates coated with ploy-lysine or collagen. Additionally, with a short, low-speed centrifugation, we observed a uniform distribution of our cells of interest across the bottom of the plate well. Conversely, we have observed that microbe cell samples suspended in complex growth media do not adhere well to the Syntrix-25-coated plates, most likely due to non-specific media components blocking sites of adherence.

Having determined a method to adhere our microbial cells of interest to a micro-titer plate surface, we next attempted to use HCA methods for the enumeration of a specific microbe as a proof of concept. Our initial experiments focused on a single strain of interest, a *Bradyrhizobium japonicum* strain used extensively in our laboratories. In a direct comparison between two separate cell counts taken from the same fermentation, we demonstrated comparable cell counts between traditional CFU/mL methods and counts obtained using direct HCA- observation approaches (Fig 1B and Table 1). Inclusion of propidium iodide (PI) or SYTOX Orange allowed for gating and removal of non-viable cell phenotypes from the enumeration of the total viable cells. We also noted that SytoBC stained a large proportion of the total cell population, but not 100% of the cell population when we used freshly thawed samples for testing as opposed to the cell samples taken directly from culture. To verify total cell staining coverage, we overlaid images from the fluorescent FITC channel and a bright-field image, which allowed us to identify a sub-population of the freshly thawed *B*. *japonicum* that remained resistant to staining with SytoBC alone. Inclusion of the general DNA-binding dye DAPI allowed us to capture the full population of objects (S1 Fig). Upon closer statistical examination of our final cell counts, comparison of the automated INCell enumeration method to the traditional CFU/mL measured sample demonstrated no significant difference based on two different tests of significance using a least squared difference of means Tukey test and a one-way ANOVA test, both performed in JMP (version 14.1) software (Table 1). Both methods demonstrated reasonable coefficient of variance (9.0% for the HCA method vs. 14.2% for the CFU/mL method), indicating good reproducibility for both methods. The ability to consistently enumerate this specific microbe with HCA when compared to traditional plating methods resulted in a dramatic improvement in our processes. The traditional CFU/mL enumeration assay for *B*. *japonicum* requires a 5–10 days incubation for quantifiable colony formation, and is much more labor intensive than the HCA methodology.

**Table 1:** *B*. *japonicum* enumeration results, traditional CFU/mL assay format compared to high content methods.

	HCA results	CFU/mL results
**Average viable cells/mL (n = 6)**	4.1 x 10^9^	3.5 x 10^9^
**Standard deviation**	3.7 x 10^8^	5.0 x 10^8^
**Percent coefficient of variance**	9.0	14.2

Encouraged by these initial enumeration results on *B*. *japonicum*, we next applied our HCA methods to the quantification of a set of diverse microbes to understand how well our object detection algorithms would function when applied to varied morphologies obtained from a variety of genera tested within the same sampling set. We also added an additional enumeration method to this comparison. Flow cytometry has been used for the quantification of various microbes[20], and we have used this technology for enumeration within our labs as well. Even though there are well known and reported issues with variability associated with the classic CFU/mL assay, it is traditionally held up as the gold standard for microbial enumeration[21]. Our experiment was based on a three-way comparison of flow cytometry, CFU/mL and our HCA enumeration methodologies for enumeration of a diverse set of microbes. verage counts/mL results for 21 genera, including both Gram-negative and Gram-positive, are presented (Fig 1C). We were unable to collect any data from the three *Streptomyces* samples included in the data set using flow cytometry, most likely due to the hyphae-like morphology of this species clogging the flow cell. Removing those three strains from the final dataset, counts obtained by CFU/mL and INCell are positively correlated (Pearson correlation coefficient 0.49, p-value 0.038), but there is no significant correlation between counts obtained by flow cytometry and either INCell or CFU/mL across a varied pool of 21 different microbial strains. What seems clear from this analysis is that both flow cytometry and HCA imaging methods can return enumeration numbers but can vary between them. We did not examine reproducibility with this experiment, but that would be the logical next step to further examine these methods for overall robustness.

**Fig 2:**
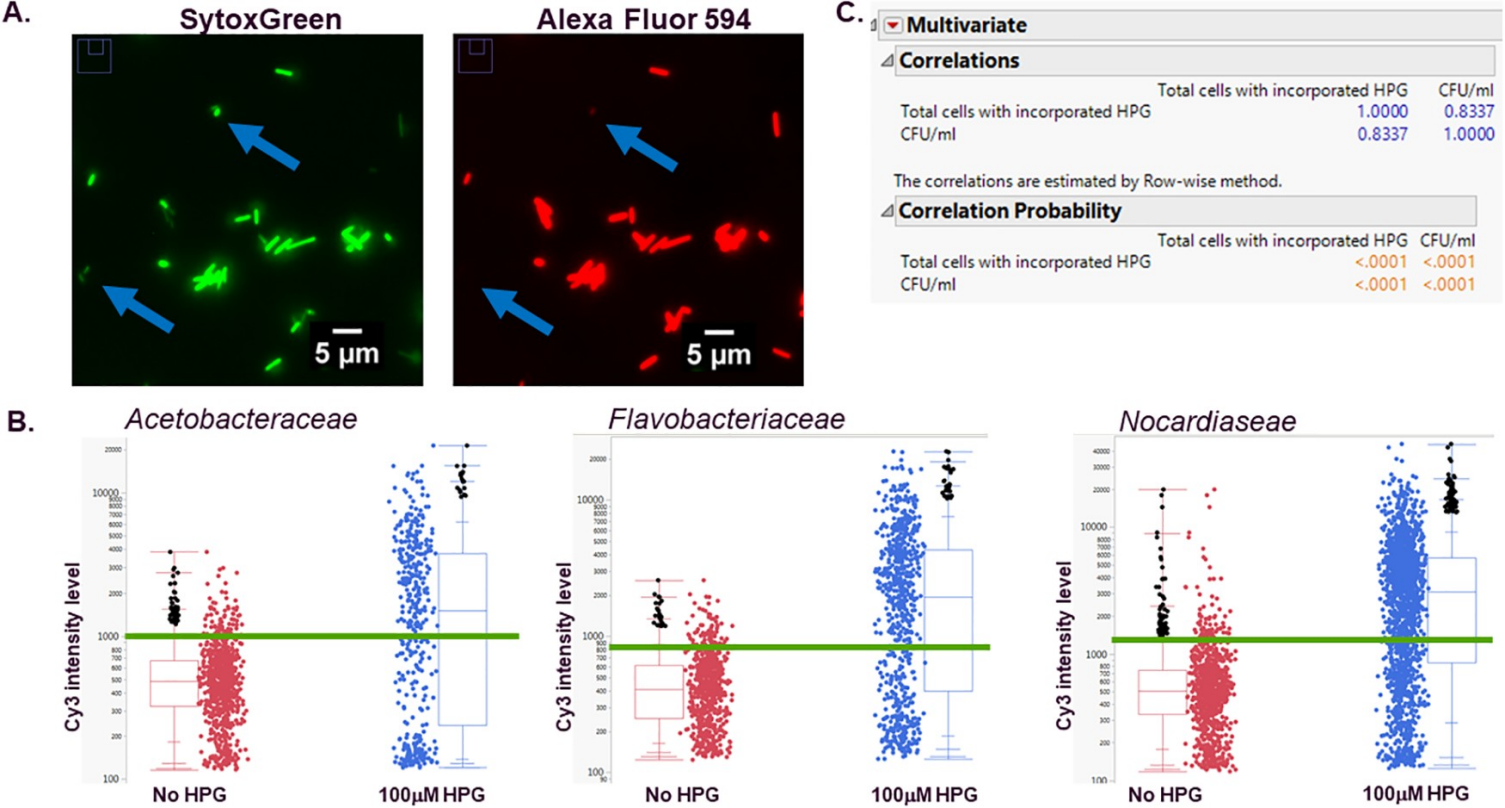
HCA protein synthesis viability determination. Two-color images of microbes incorporating HPG (A). Total DNA was stained with SYTOX Green and detected in the FITC channel, synthesized protein with incorporated HPG and ligated Alexa Fluor 594 was detected in the Cy3 channel. Arrows indicate cells positive for DNA staining, but negative for HPG incorporation. Three representative data sets demonstrating the ability to separate individual cell populations into viable and non-viable populations based on a control sample without the artificial amino acid HPG compared to HPG-containing samples. Green lines demonstrate the approximate Cy3 intensity value used to determine HPG incorporation as opposed to autofluorescence. Individual object data are represented by single dots, summary data is reported with box and whisker plots (B). Statistical analysis of enumeration results from 52 (largely *Bacillus*) samples determined by HCA measured cell viability via CLICK chemistry and CFU/mL assays (**C**).

Next, as we expanded the potential of using HCA for microbial characterization, we focused on the determination of microbe viability and alternatives to membrane potential dyes. Various methods are used in mammalian cell culture to measure cell viability[22]. Typically, for microbes, CFU/mL plating is used as both a measurement of viable cells and cell enumeration, based on the understanding that a single viable cell will give rise to visible colonies that can be counted. We attempted to develop a high content, high-throughput method to simultaneously enumerate as well as determine the number of viable cells to streamline workflows and reduce data turn-around time lines. We took advantage of the ability of microbes to incorporate an artificial amino acid, in this specific instance L-homopropargylglycine (HPG), into the pool of total cellular protein to allow for the identification of cells actively synthesizing protein. These proteins were then detected by labeling the incorporated HPG via ligation of a Alexa Fluor 594 molecule by copper-mediated chemical reaction, commonly referred to as a CLICK Chemistry reaction [23]. Finally, the labeled proteins could then be identified with high-content imaging. There is a distinct advantage to using a biochemical process, in this case protein synthesis, as a marker of viability, as opposed to detection of a dye such as PI or SYTOX Orange whose activity is based on viable cellular exclusion. All processes were performed in multiwell plates, using various automated liquid-handling equipment and image acquisition, highlighting the high-throughput capability of the process. Data analysis required analyzing flluorescent images, by first identifying the individual cells by staining their DNA content with SYTOX Green, and then determining the level of incorporated HPG by measuring the level of Cy3 intensity based on the amount of Alexa Fluor 594 ligated to the cellular pool pf proteins (Fig 2A and 2B). For this analysis, the third channel DAPI data was ignored.

Again, using a diverse set of microbes as our subject pool, we compared agar plate-based CFU/mL data to those obtained with HCA and HPG incorporation. Comparison of results from the two methods indicated that counts were highly correlated to each other (Pearson correlation coefficient 0.83, p-value <0.0001), indicating the HCA method described is robust and suitable for enumeration and detection of viable microbes, when CFU plating is used as the basline. We also noted that out of 75 total microbes tested, 6 *Actinomyces*, 5 Gram-negative strains, and 6 *Bacillus* did not grow on agar plates, but we were able to detect viable cells using the HCA methodology. Alternatively, we also identified two *Bacillus* strains that did not indicate viable cells using the HPG incorporation method, but did show growth on agar plates. Finally, one Gram-negative strain, one *Bacillus* strain, and two *Actinomyces* strains were undetected with either method, suggesting the original sample inoculation contained no viable cells. These results indicate the HCA method may improve enumeration for microbial strains that are difficult to grow on agar.

Comparing total viable cells obtained with our HCA method to CFU/mL plating, we demonstrated a significant correlation between the two methods. We excluded samples that demonstrated no growth or detectable protein synthesis from the final sample analysis, removing 23 samples from the final data set and lowering the final comparison to 52 total samples. With this final comparison, we can conclusively state that HCA methods of microbe enumeration and viability determination based on total protein synthesis are comparable to traditional CFU/mL assays.

**Fig 3:**
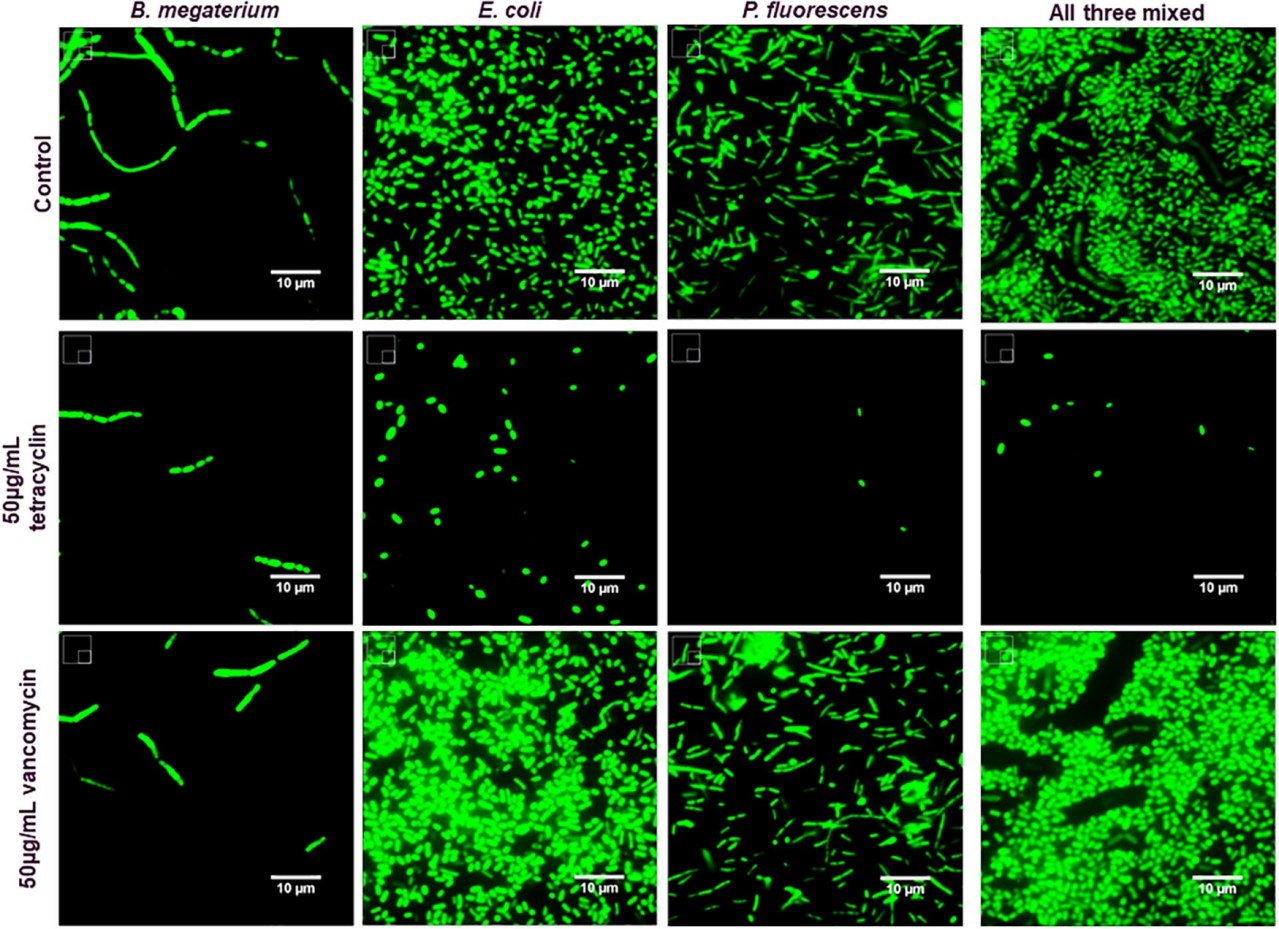
Representative images of microbes treated with antibiotics. Representative images of control and antibiotic-treated microbes stained with the DNA dye SYTOX Green are presented. Images were normalized for veiwing using ImageJ within each genus for comparison, mixed microbe samples were normalized to each other.

As an additional proof of concept that HCA analysis methods could be applied to complex microbial samples, overnight antibiotic treatments were performed with the Gram-positive strain *B*. *megaterium*, and two Gram-negative strains, *Escherichia coli* K12, and *Pseudomonas fluorescens*. We also mixed the three strains together before antibiotic treatment. After fixation and staining with SYTOX Green, we applied the same individual object detection, measurement, and enumeration techniques as in the previous discussion. We were easily able to identify anti-proliferative effects from the three antibiotics, along with indications of drug specific effects on microbial cellular morphology for each species (Fig 3 and S2 Fig). Treatment with vancomycin, which mainly targets cell wall synthesis, demonstrated potent Gram-positive targeted effects[24,25],but less effect against the Gram-negative strains. Tetracycline, which binds the 30S ribosomal subunit[26], demonstrated activity against all three strains, but appeared to be most potent against the Gram-negative strains, based on observation of total objects detected in each sample as compared to control.

**Fig 4:**
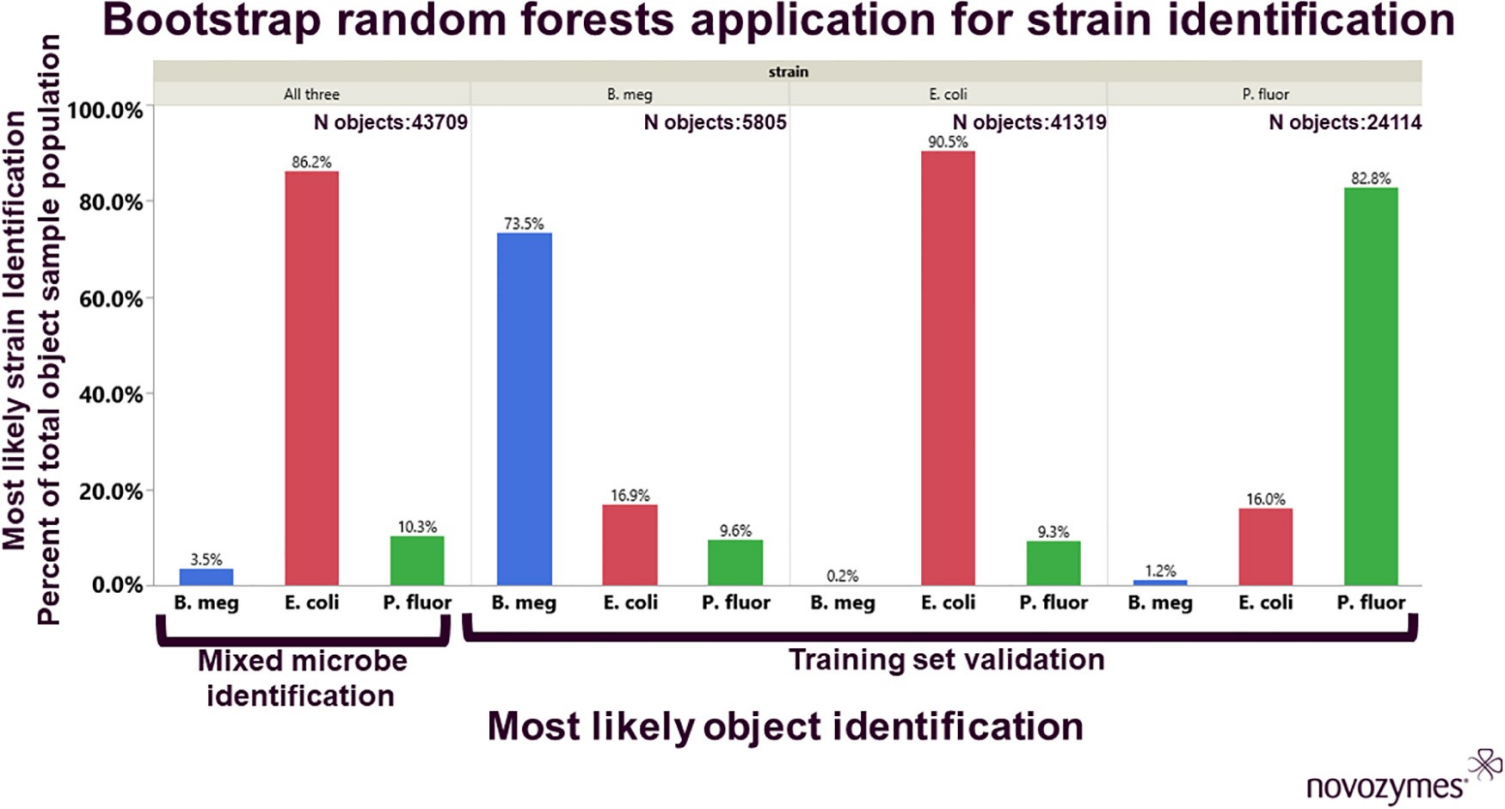
Object feature based strain identification in mixed culture. Data represented is based on the Bootstrap random forest analysis of the total objects (indicated by N) for each microbe species tested. First, subsamples of data from each species in the un-treated control group were used to train the algorithm. Following training, accuracy of the algorithm was determined by re-analysis of all data from each microbe. Data is presented as the percent of objects identified within each training set, so that correctly identified microbes match the identified sub-group, mis-identified microbes do not. Once we validated the rate of correct identification, the algorithm was applied to the mixed-microbe sample object data set, values returned correspond to the percent objects identified as either *B*. *megaterium*, *E*. *coli*, or *P*. *fluorescens*.

As a final test of robustness, we used the morphological feature data from the control images, for each individual strain tested with antibiotic treatment, as training sets for Bootstrap random forest analysis. This analysis was partitioned into 60%/20%/20% training/test/validation sets and run with default model building settings in JMP Pro (version 14). We then used the resulting model to classify the individual objects from the untreated mixed cultures. This machine learning analysis allowed for the identification and quantification of the individual microbe species within the mixed culture sample (Fig 4). For all three strains, applying the model to the full single-microbe datasets showed positive identification rates of 73–90.5% of the individual objects for each strain. False rates of identification ranged from 0.2–16.9%. Then, when applied to the data from the untreated mixed strain samples, the Bootstrap random forest model indicated that *E*. *coli* growth had dominated the other two test strains, with 86.2% total of the population being identified as this strain. Visual inspection of the sample images supported the low percentage of *B*. *megaterium* makeup within the mixture called by the model analysis, but further effort to confirm the ratio of *E*. *coli* to *P*. *fluorescens* will be undertaken and reported in a future manuscript, as will analysis of antibiotic strain specificity within mixed microbe sample treatments.

These early results present the possibility of expanding HCA of microbial samples to more complex questions, such as determining effects of mixed-microbe cultures on the effectiveness of various antibiotic treatments. Our future research will further explore the utility of various machine learning and artificial intelligence approaches in the interpretation of complex data sets based on mixed-microbial cultures and responses to various environmental factors.

## Conclusions

We have demonstrated the capability of HCA techniques and data analytics to record morphological characteristics of microbes, expanding the functionality of HCA from mainly mammalian cell culture treatments to the field of microbiological analysis. The described HCA techniques enable successful enumeration and viability determination of diverse classes of microbes, help identify cells that may be classified as viable but not culturable, and allow for the classification of mixed-microbe populations using machine learning modelling. Taken together, these results support the wide spread adoption of HCA methodologies in large-scale microbiological applications.

We have documented reduced time frames for the determination of microbe viability as compared to traditional agar plate enumeration methods. The multiplex sampling methods used here greatly reduced the amount of plastics and reagents typically used for traditional CFU/mL enumeration methods. A single 384-well plate produces more HCA data than the comparable stack of 384 petri dishes, does not need the resources and time necessary to prepare and enumerate those petri dishes, and reduces disposal needs. A practical consideration is the benefit of adapting protocols to liquid handling robotics, and the ability to save raw data as a digitalized image, providing long-term data integrity, unlike a biological agar plate sample. Most of the CFU/mL plating techniques described here are standard for all microbiology laboratories, but these HCA processes are particularly amenable to many large-scale microbiome projects currently underway. Additional benefits could be discovered if these HCA techniques are eventually further refined and validated for use in quality assurance and quality-control regulatory laboratories.

Finally, we show that combining machinelearning approaches with HCA generated data, an approach now commonly used in drug discovery, can be done in a similar manner with microbial sample datasets. This method allows for identification of changes in microbial morphological characteristics in response to various treatments and culture conditions. Recently, Zoffmann et. al. published a comprehensive description of various machine-learning techniques applied to imaged bacteria samples treated with compounds for the purpose of discovering novel antibiotic compounds[27]. We have utilized a similar approach to identify morphological changes of three different microbes in response to two antibiotics with different modes of action. Additionally, we have shown that machine learning could allow for identification of population members of mixed microbe samples, demonstrating the potential of testing numerous microbes simultaneously for mixed population responses, or potentially biofilm formation or degradation in response to treatment. In both cases, application of machine learning techniques has enhanced our abilities to test complex theories and return robust, annotated data sets to drive research, reduce data turn-around timelines, and ultimately enhance the ability of microbial discovery research to progress at an increasing pace.

## Supporting information

S1 Fig Stain coverage of *B*. *japonicum*. Example images of *B*. *japonicum* enumeration samples demonstrating variable SytoBC staining, uniform DAPI staining, and SYTOX Orange viable staining. Images were adjusted for color and zoom level using ImageJ.
(TIF)

S2 Fig Representative HCA data from antibiotic treatment of microbes. All data presented are from the same sample dilution to maintain data consistency. Microbes were treated in triplicate wells, and each well was imaged in duplicate for a total of n = 6 sample wells. Error bars indicate the standard error of all objects represented. Average individual object counts per well of treated microbe samples from the 1:250 sample dilution are presented (A). The morphological characterization of roundness, measured as Form Factor, was averaged for all object data collected from *P*. *fluorescens*, 1:50 dilution samples. Form Factor is scaled from 0–1, with 1 being a perfect circle, and 0 being a straight line. Total object area for treated *B*. *megaterium*, 1:250 sample dilution, was determined. Size variance was shown to be dose and compound-class dependent (C). SYTOX Green staining intensity levels, generally associated with DNA content or condensation were measured in treated *E*. *coli* 1:250 sample dilution, with effects on staining intensity only noted from tetracycline treatments.
(TIF)

S1 Table CFU/mL data vs HCA data generated from *B*. *japonicum* samples. All viable cells/mL values are the mean calculated from triplicate determinations from at least 3 individual sample dilutions that displayed a dilutional linearity R^2^ response >0.95.
(JMP)

S2 Table Three-way enumeration comparison. Direct comparison of three different cell enumeration methods, traditional CFU/mL plating, Flow cytometry, and HCA.
(JMP)

S3 Table Click viability vs. CFU/mL. Direct comparison of viable cell number determination using total cellular protein synthesis detected via HCA compared to CFU/mL plating methods.
(JMP)

S4 Table Antibiotic analysis results. Individual object data set collected from treatment of three different microbe species, and a mixed species sample, treated with tetracycline or vancomycin. Morphological data for each individual identified object is described in the individual column headers.
(JMP)

S5 Table GE Developer script for enumeration of *B*. *japonicum*. Example of the detection algorithm script developed within GE Developer software and applied to images of B. *japonicum* for determining sample viability enumeration. An intensity cut-off of 100 was used for PI-stained samples, and a 1000 intensity cut-off was used for samples stained with SYTOX Orange.
(TXT)

S6 Table GE Developer script for analysis of 3 color microbe images. This script was used to analyses images obtained from antibiotic treatments of microbes and determination of microbe viability via protein synthesis detection.
(TXT)
