# Supplementary figures and images for: Use of high-content analysis and machine learning to characterize complex microbial samples via morphological analysis
Source: PLoS One. 2019 Sep 23;14(9):e0222528. doi: 10.1371/journal.pone.0222528 (PMC6756541; doi:10.1371/journal.pone.0222528)

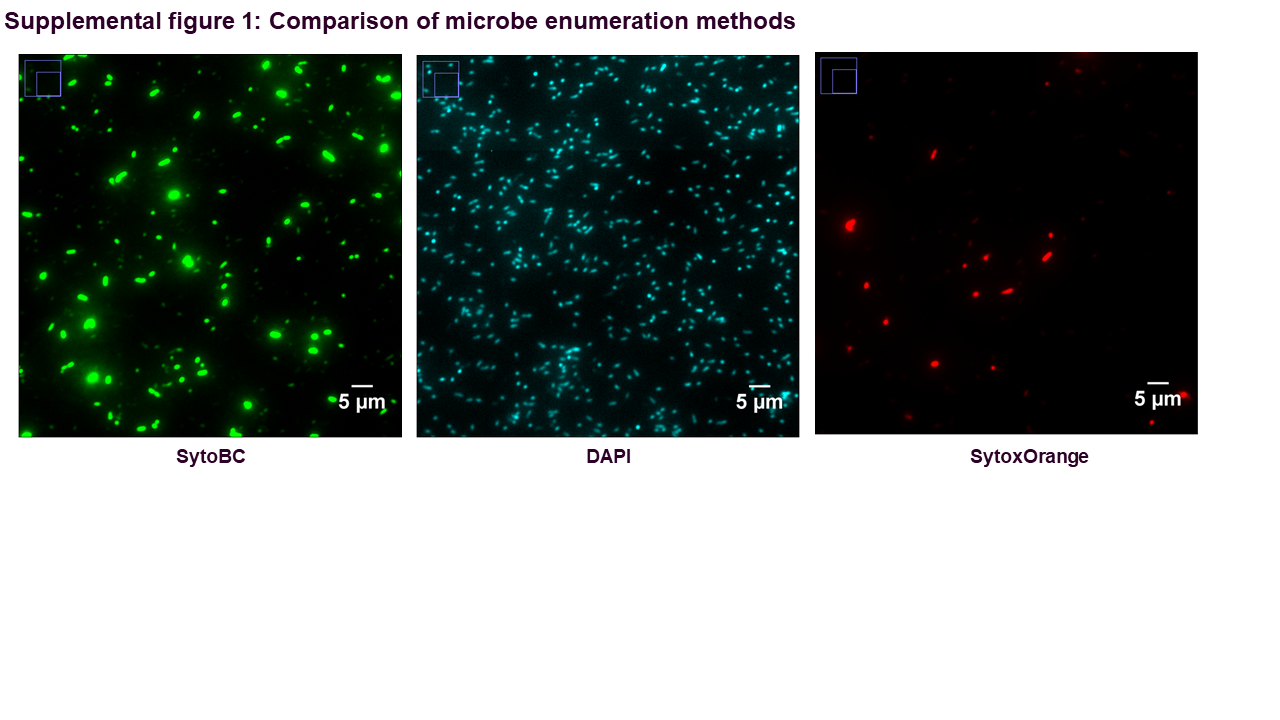

Supplement: S1 Fig — Example images of B. japonicum enumeration samples demonstrating variable SytoBC staining, uniform DAPI staining, and SYTOX Orange viable staining. Images were adjusted for color and zoom level using ImageJ. (TIF) [file pone.0222528.s001.TIF]

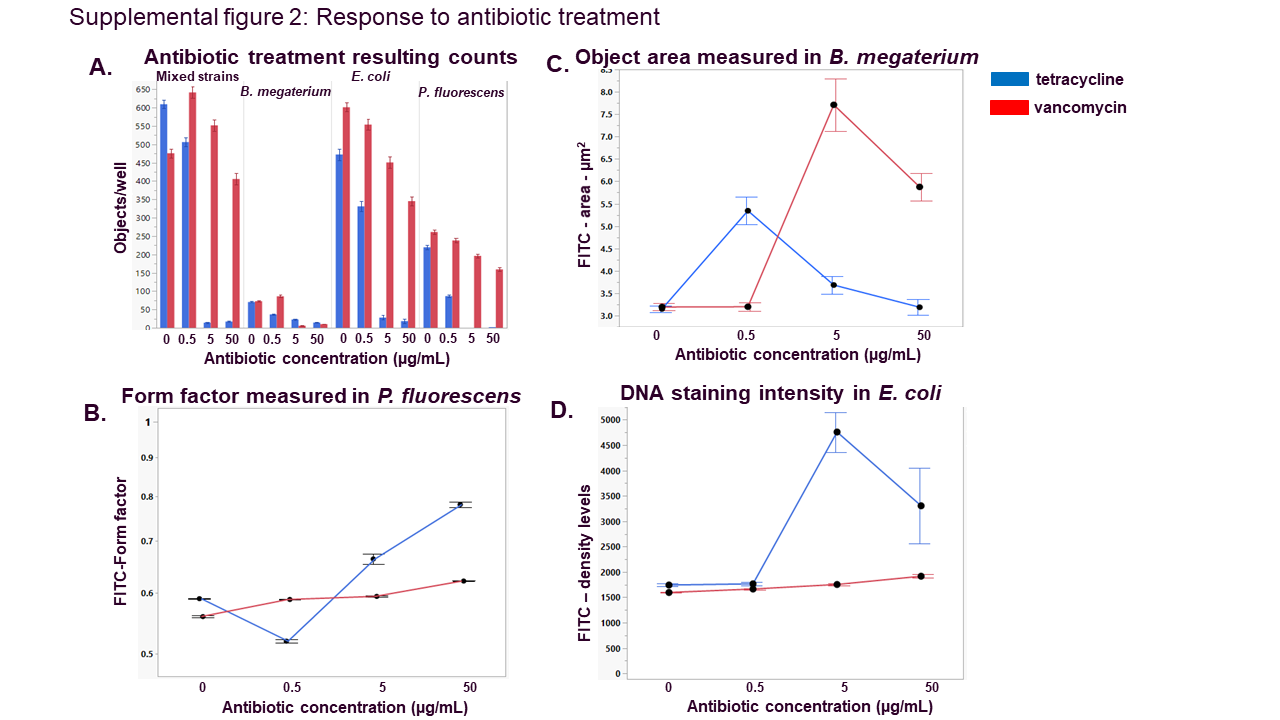

Supplement: S2 Fig — All data presented are from the same sample dilution to maintain data consistency. Microbes were treated in triplicate wells, and each well was imaged in duplicate for a total of n = 6 sample wells. Error bars indicate the standard error of all objects represented. Average individual object counts per well of treated microbe samples from the 1:250 sample dilution are presented (A). The morphological characterization of roundness, measured as Form Factor, was averaged for all object data collected from P. fluorescens, 1:50 dilution samples. Form Factor is scaled from 0–1, with 1 being a perfect circle, and 0 being a straight line. Total object area for treated B. megaterium, 1:250 sample dilution, was determined. Size variance was shown to be dose and compound-class dependent (C). SYTOX Green staining intensity levels, generally associated with DNA content or condensation were measured in treated E. coli 1:250 sample dilution, with effects on staining intensity only noted from tetracycline treatments. (TIF) [file pone.0222528.s002.TIF]
